# Unusual Genital Wart Lesions: A Case Series on Angiokeratoma of Fordyce

**DOI:** 10.7759/cureus.56757

**Published:** 2024-03-23

**Authors:** Anannya S, Sharada RG, Afthab Jameela Wahab

**Affiliations:** 1 Department of Dermatology, Saveetha Medical College and Hospital, Saveetha Institute of Medical and Technical Sciences, Saveetha University, Chennai, IND

**Keywords:** vascular, fordyce, vulva, scrotum, unilateral, angiokeratoma

## Abstract

Angiokeratoma is a vascular cutaneous disorder that is generally asymptomatic and presents with multiple dark red to blue or black papules over the skin. The prevalence of angiokeratoma increases as the age increases and it is more common after third and fourth decades of life. There are different types of angiokeratoma which may be localized forms (angiokeratoma of Mibelli, angiokeratoma circumscriptum, solitary angiokeratoma, and angiokeratoma of the scrotum or vulva) or diffuse variant (angiokeratoma corporis diffusum). Here, we report a series of five rare cases of angiokeratoma of Fordyce, of which two cases had vulval involvement and one case showed lesions on unilateral scrotal wall which was unusual.

## Introduction

Angiokeratoma of Fordyce is the most common type among the five variants of angiokeratoma. It is seen in around 0.16% of the general population and on the whole accounts for 14% of all angiokeratomas [1]. It is regarded as a degenerative disorder and there is evidence that local venous hypertension plays a part in its development [2]. It becomes more frequent with increasing age. Clinically, it presents as small 1-4 mm bright red vascular papules on the scrotum as early as late adolescence. With increasing age, they become darker, larger, and more numerous. There may be itching, pain, burning sensation, bleeding, or soreness [3]. The lesions are usually present over the scrotum and identical lesions may also occur on the penis, upper thighs, and in the groin. Liquid nitrogen cryotherapy, laser ablation, or diathermy can be used to treat symptomatic lesions. In our case series, we report as rarely encountered findings, a unilateral variant in a male and the sudden pregnancy-associated worsening of vulval lesions with complete resolution post-partum in a female. This adds to the existing literature and aids in diagnosing patients with unusual presentations.

## Case presentation

**Figure 1:**
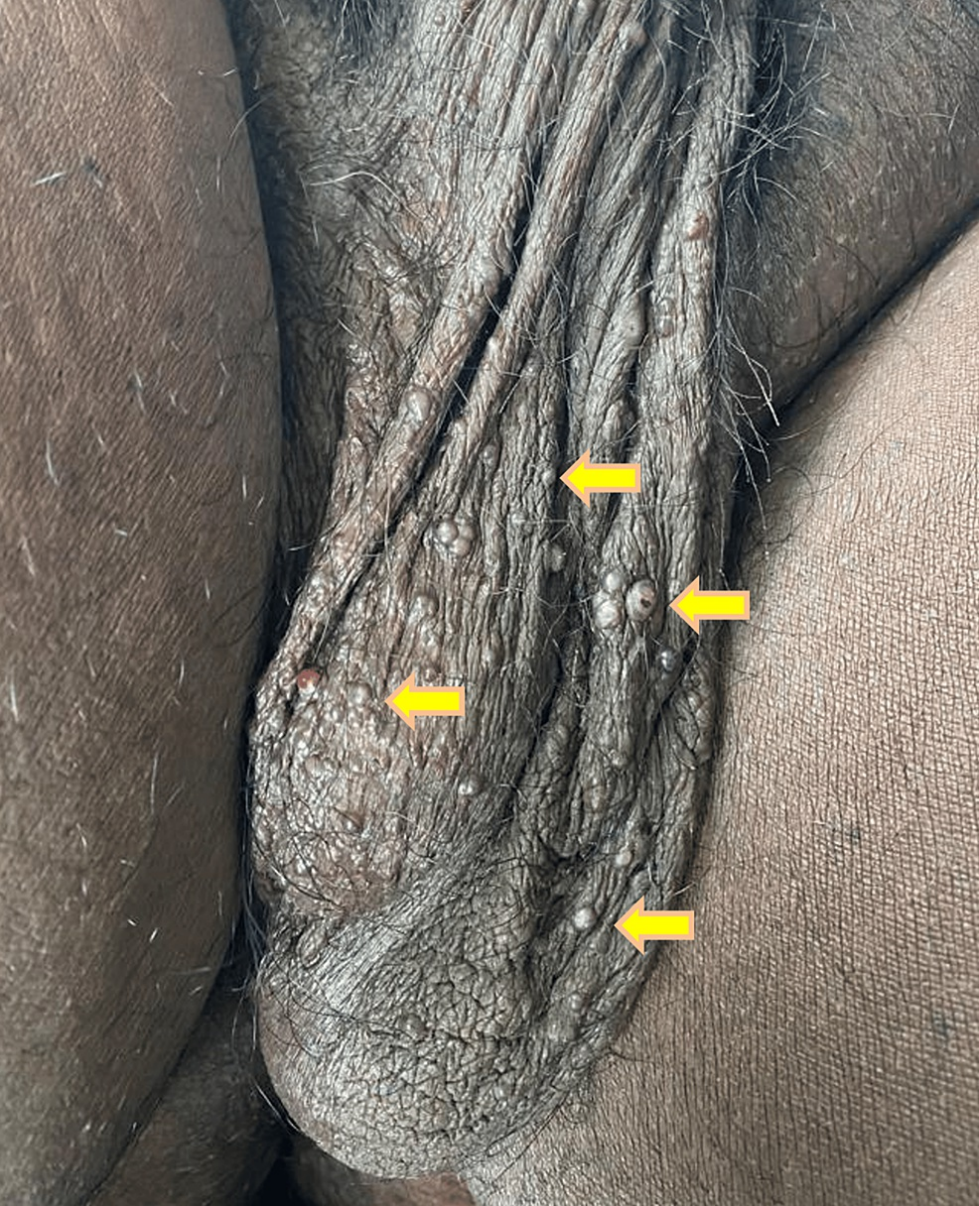
Multiple dusky-red-brown hyperkeratotic papules clustered over the scrotal skin (yellow arrowhead)

**Figure 2:**
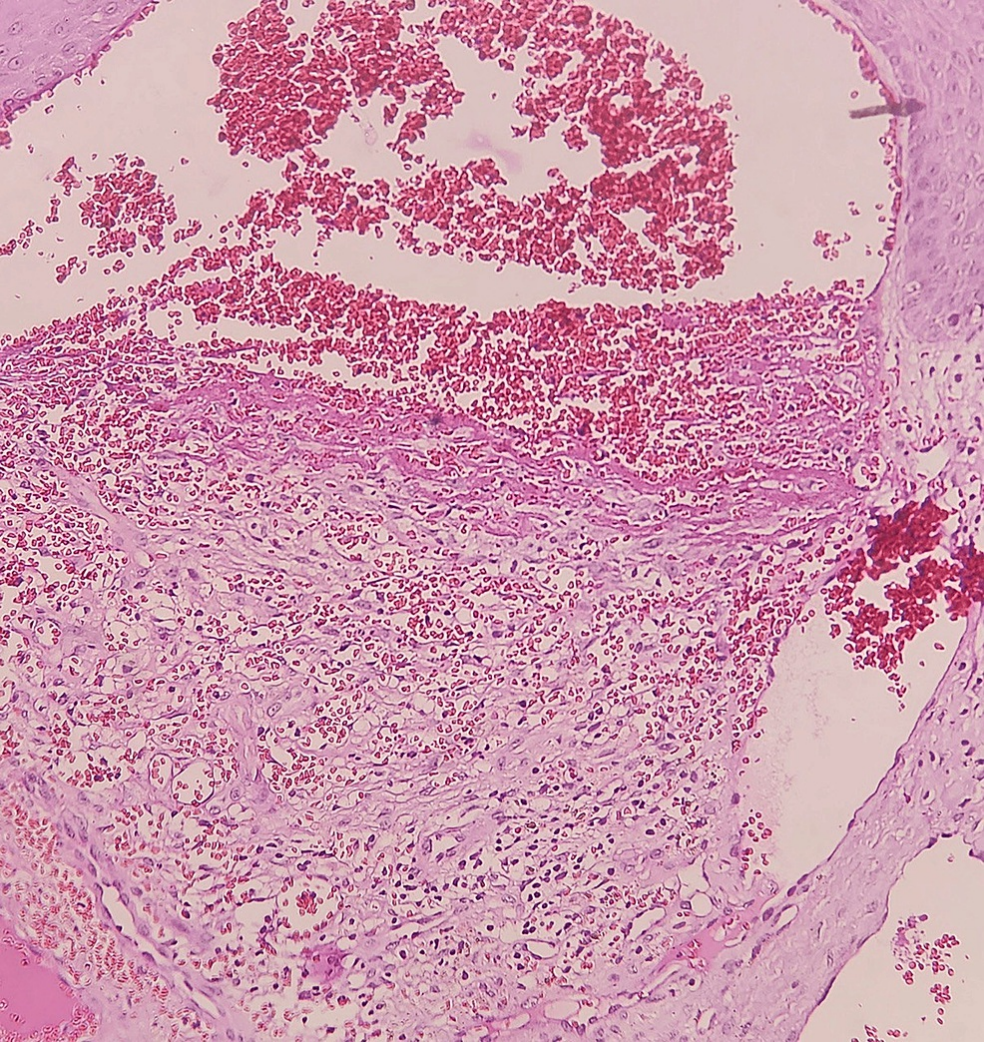
Hematoxylin and eosin stained slide at 40x magnification showing dilated blood vessels in the papillary dermis

Case 1 was a 45-year-old male who presented with multiple raised lesions over the scrotum (Figure 1) since five months. Examination revealed multiple dusky red-brown hyperkeratotic papules clustered over the scrotal skin which bled on touch. On histopathological examination, there was hyperkeratosis, acanthosis and the underlying papillary dermis showed many ectatic dilated vascular spaces encircled by the epidermis and few vascular spaces showed thrombi. The superficial dermis showed few vascular spaces (Figure 2) and the subcutaneous tissue appeared unremarkable.

**Figure 3:**
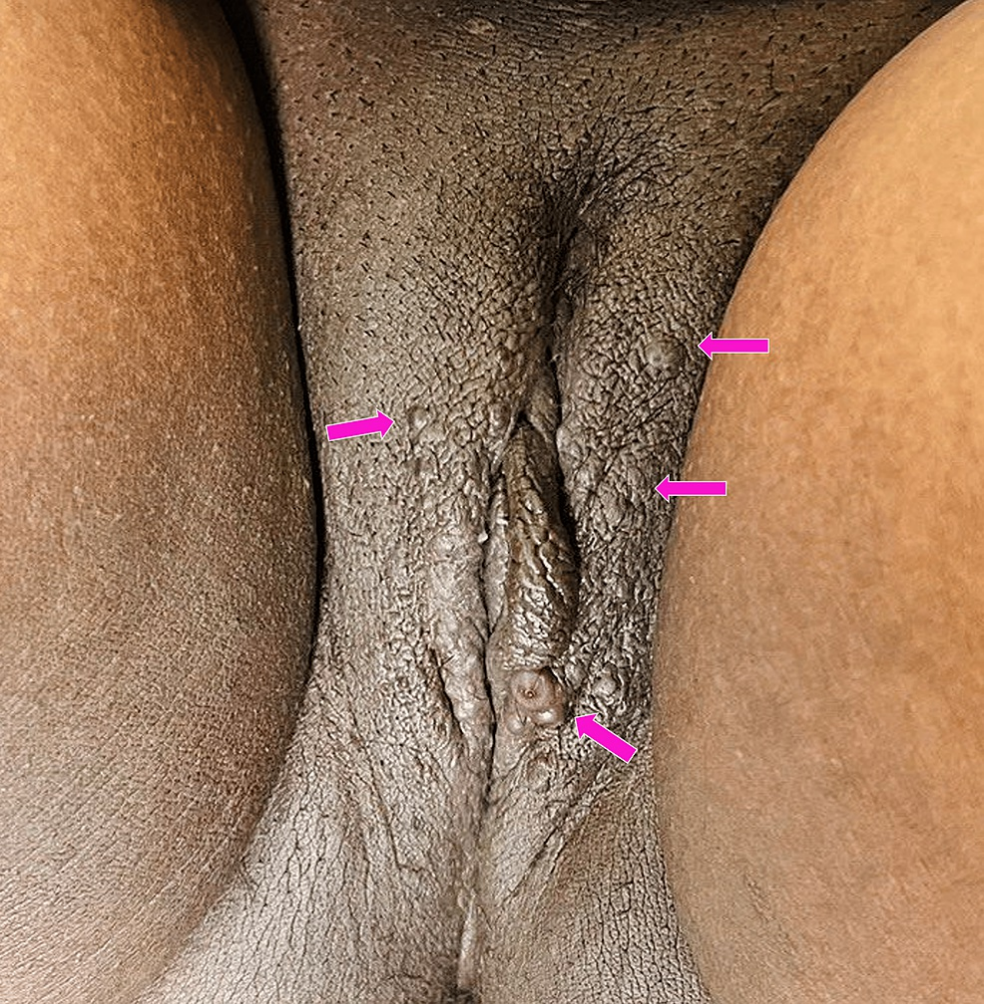
Multiple skin-colored papules over vulva in a 34-year-old primigravida which bled on touch (pink arrowhead)

Case 2 was a 34-year-old female, primigravida (gestational age - 31 weeks) who presented with multiple skin colored raised lesions over the genital region (Figure 3) since one month with a history of rupture and bleeding of few lesions since two days. Similar lesions had appeared occasionally over the past nine years showing an increase in size and number during menstruation and regressing spontaneously. She was a known case of diabetes mellitus and hypothyroidism. On examination, multiple pinkish-brown papules were seen over the labia majora. A biopsy was not done as the patient was not willing.

**Figure 4:**
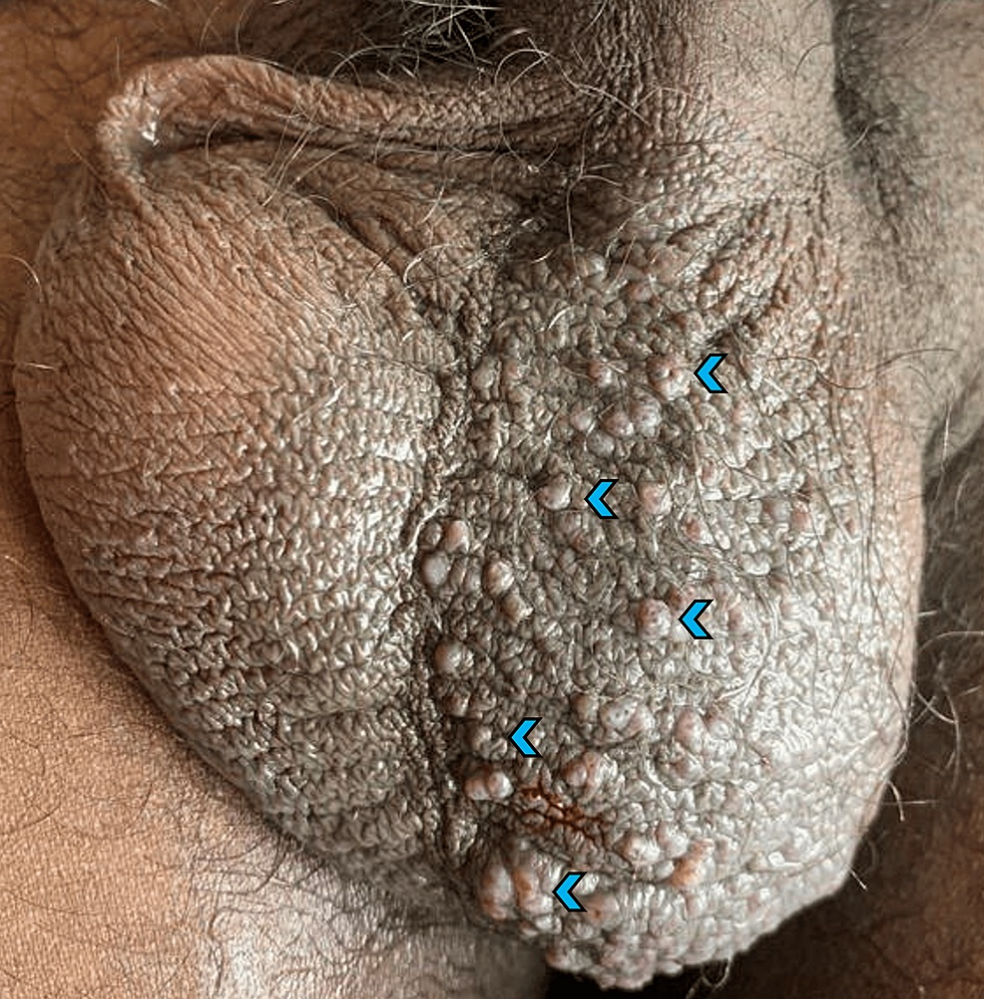
Multiple small pinkish papules over the left side of the scrotum (blue arrowhead)

**Figure 5:**
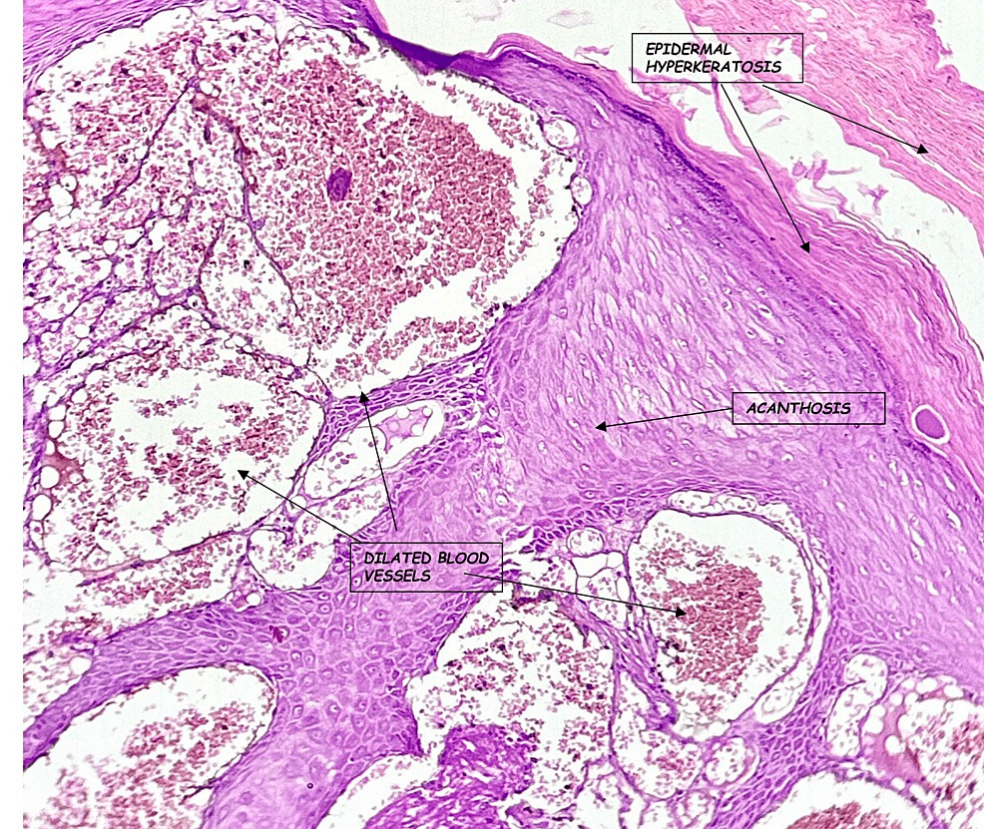
Histopathological examination shows ectatic superficial dermal vessels, an overlying hyperkeratosis, and epidermal collarette (H&E, 10x) H&E: hematoxylin and eosin stain

Case 3 was a 50-year-old male who presented with complaints of multiple small pinkish raised lesions over the left side of the scrotum (Figure 4) since four months. Examination showed multiple tiny skin-colored papules over the left side of the scrotum which bled on touch. A histopathological examination (HPE) of the skin lesions showed many ectatic superficial dermal vessels, an overlying hyperkeratosis, and an epidermal collarette (Figure 5).

**Figure 6:**
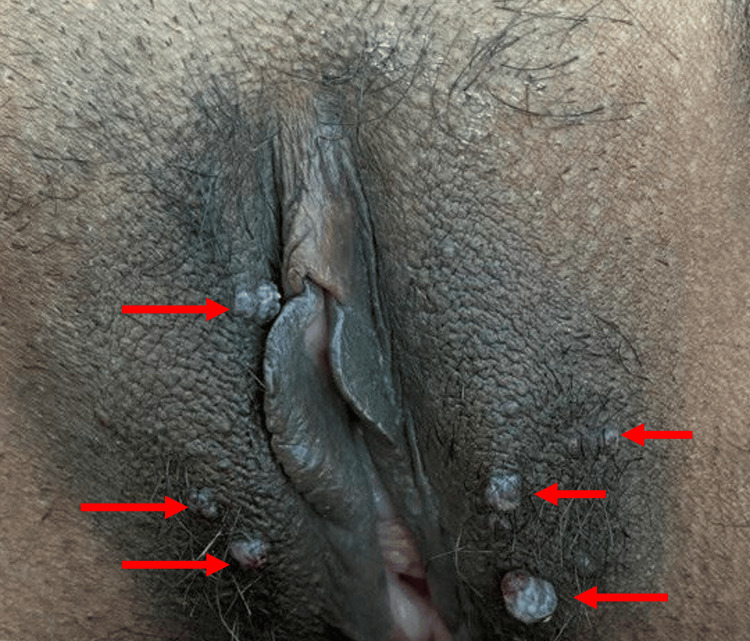
Multiple, rounded, hyperpigmented, erythematous papules 1-5 mm over the labia majora (red arrowhead)

**Figure 7:**
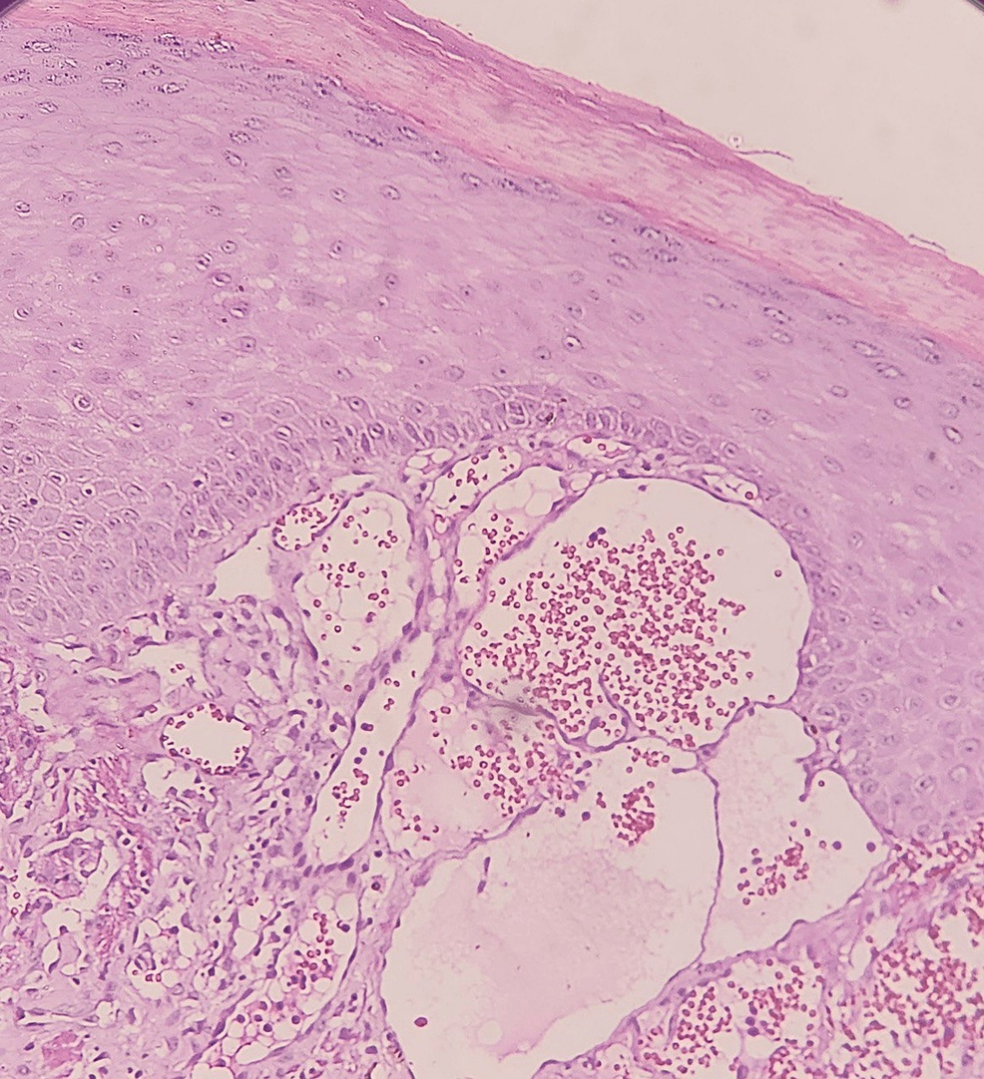
Histopathology image showing dilated vascular spaces within the upper dermis, with overlying epidermal hyperkeratosis (H&E, 40x) H&E: hematoxylin and eosin stain

Case 4 was a 36-year-old female who presented with painless dark raised lesions in the vulva (Figure 6) for the past three months. It was insidious in onset and gradually progressive in size. On examination, multiple, rounded, hyperpigmented, erythematous papules 1-5 mm over the labia majora of the vulva were noted which bled on touch. Histopathology showed dilated vascular spaces within the upper dermis, with overlying epidermal hyperkeratosis (Figure 7).

**Figure 8:**
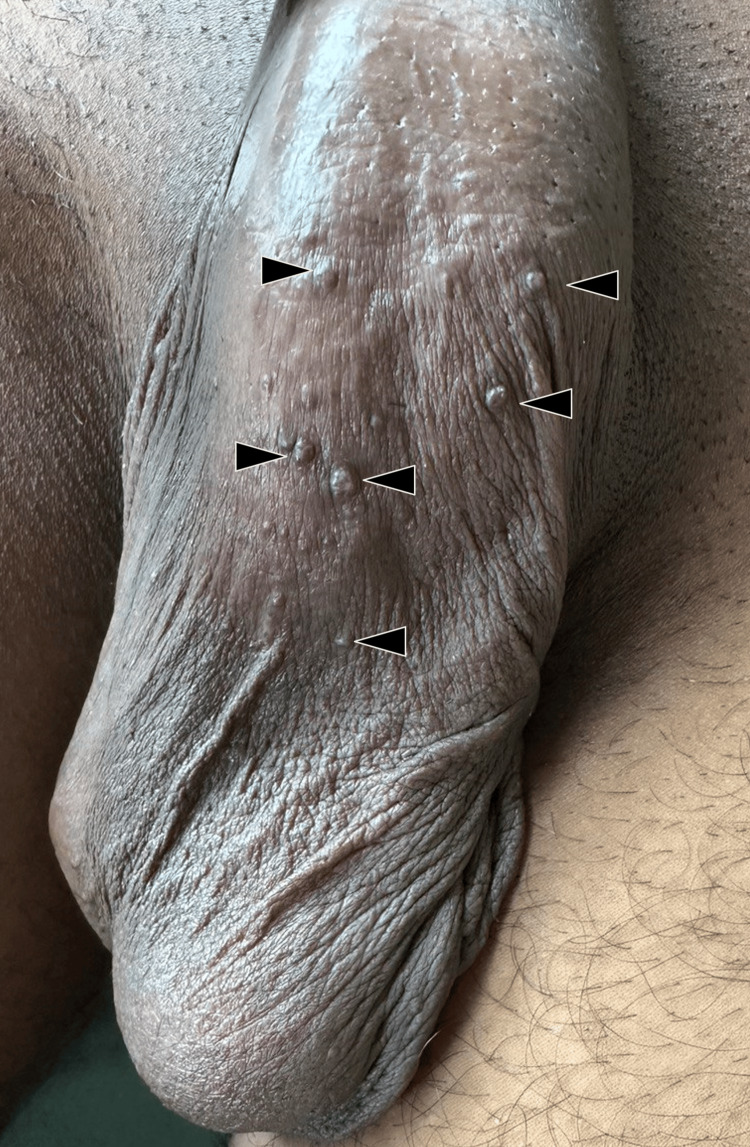
Small reddish brown papules over the scrotal skin (black arrowhead)

**Figure 9:**
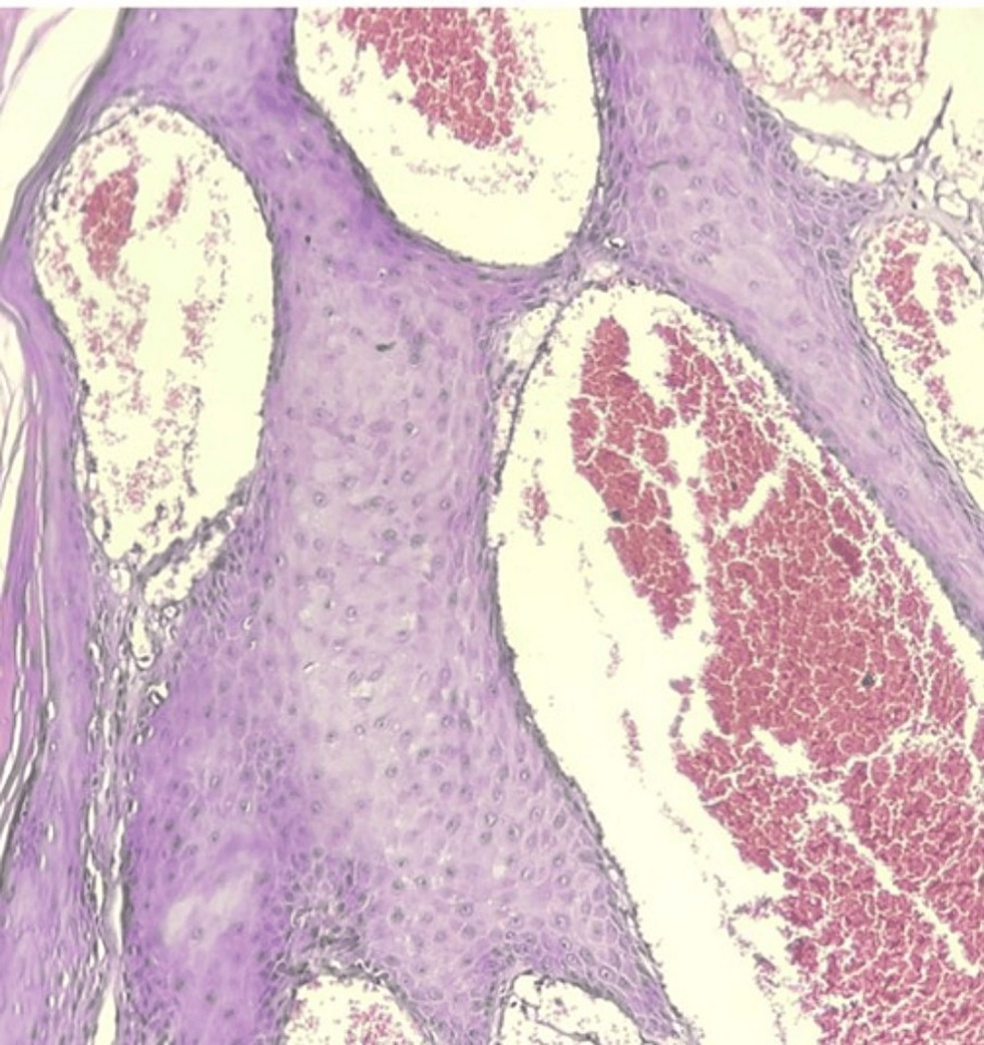
Ectatic superficial dermal capillaries

Case 5 was a 42-year-old male who presented with multiple painless swellings in the scrotum (Figure 8) since two years with a history of bleeding on touch. Examination revealed numerous small reddish brown papules that bled on touch, with no evidence of hernia or hydrocele. Histopathology showed multiple dilated vascular spaces (Figure 9).

## Discussion

Angiokeratomas are well-circumscribed vascular lesions with superficial vascular ectasia and hyperkeratosis [4]. Among the five types, angiokeratoma of Fordyce is the most common variant. In 1896, John Addison Fordyce diagnosed the first case in a 60-year-old patient over the scrotal skin [5]. It may arise in the second or third decade but is most commonly seen in the older age. The most widely accepted hypothesis behind the development of angiokeratoma is venous pressure. Increased venous pressure or vascular malformation results in the disintegration of elastic tissue in the blood vessel wall which results in telangiectasia [6]. Prolonged irritation and mechanical trauma are the other predisposing factors of angiokeratoma. Friction and irritation cause the expression of matrix metalloproteinase-9 enzyme in the epidermis, which stimulates epidermal reactions such as acanthosis and hyperkeratosis [7]. The underlying pathology of varicocele, thrombophlebitis, or inguinal hernia may increase the venous pressure which stimulates the production of genital angiokeratomas. Increased venous pressure is noticed in pregnancy, vulval varicosity, post-partum, and post-hysterectomy in women, leading to angiokeratoma. Clinically, the lesions are usually red or black in color, single or multiple, and arise along superficial vessels. They may be associated with varicocele, thrombophlebitis, and inguinal hernias.

The histologic findings are essentially the same in all types of angiokeratoma and consist of hyperkeratosis, acanthosis, and elongation of the rete ridges, with numerous, dilated, thin-walled, congested capillaries mainly in the papillary dermis [8]. Similarly, in this case series, consistent findings of hyperkeratosis, mild acanthosis, and pseudoepithelial hyperplasia in the epidermis and ectatic blood vessels in the papillary and reticular dermis are present.

Dermoscopy was done in case 5 which revealed multiple dilated vascular spaces as red-black lacunae with a whitish veil which was in accordance with similar case reports [9,10].

In our study, one patient had typical clinical features of angiokeratoma of Fordyce involving the scrotal skin diffusely, two female patients had vulval involvement of whom one was pregnant and the lesions increased in size and number with an increase in gestational age and the last patient had lesions on the unilateral scrotal wall which was highly unusual. Only six cases of unilateral angiokeratoma of the scrotum have been reported which is in concurrence with the studies done by Erkek et al., Bechara et al., Piqué-Duran et al., Pande et al., González-López et al., and Vinod et al. [6,11-15].

On the other hand, angiokeratomas of the vulva are relatively rare findings and the presentation of our patients mimics the description of cases previously published with asymptomatic small red papules disseminated over the labia majora by Buljan et al., Doğan et al., Samudrala et al., and Bjekić et al. [16-19].

Management includes surgical excision, curettage, radiofrequency cautery, cryotherapy, electrocoagulation, and laser ablation. Electrocautery and gentle local excision were found to be the preferred methods of intervention in many reports. Topical rapamycin has recently emerged as a treatment for angiokeratoma [20]. If the venous pressure in the scrotum is increased due to underlying pathology such as varicocele or inguinal hernia, treating the cause may reduce angiokeratoma. Other than support and assurance, treatment is not needed for most patients with angiokeratoma of Fordyce.

Treatment of our patients (cases 1, 3, 4, and 5) involved removal of lesions by radiofrequency cautery with no recurrence after a six-month follow-up except in case 2 where spontaneous resolution of lesions occurred after delivery without any intervention.

Differential diagnoses include verruca vulgaris, pigmented basal cell carcinoma, dermatofibroma, condyloma acuminatum, Spitz nevi, hereditary hemorrhagic telangiectasia, lymphangioma circumscriptum, and pyogenic granuloma.

## Conclusions

Here, we report a series of five cases of angiokeratoma of Fordyce with two patients presenting with unusual features. The rarely seen unilateral angiokeratoma of the scrotum, the sudden worsening of lesions during pregnancy, and complete spontaneous resolution post-partum in a female may add to the clinical spectrum of genital angiokeratomas. Awareness of such rare presentations may help to differentiate from other causes of genital growths including the more common genital warts.

## References

[1] Beutler BD, Cohen PR (2017). Angiokeratoma of the glans penis. Skinmed.

[2] Agger P, Osmundsen PE (1970). Angiokeratoma of the scrotum (Fordyce). A case report on response to surgical treatment of varicocele. Acta Derm Venereol.

[3] Taniguchi S, Inoue A, Hamada T (1994). Angiokeratoma of Fordyce: a cause of scrotal bleeding. Br J Urol.

[4] Requena L, Sangueza OP (1997). Cutaneous vascular anomalies. Hamartomas, malformations and dilatation of preexisting vessels. J AM Acad Dermatol.

[5] Raja Babu KK, Harinarayana P, Vijayakumar B, Rao TS, Reddy BS, Rao PS (1980). Angiokeratoma of Imperial and Helwig. Indian J Dermatol Venereol Leprol.

[6] Erkek E, Basar MM, Bagci Y, Karaduman A, Bilen CY, Gokoz A (2005). Fordyce angiokeratomas as clues to local venous hypertension. Arch Dermatol.

[7] Kobayashi T, Sakuraoka K (1998). A case of angiokeratoma circumscriptum: immunolocalization of matrix metalloproteinase (MMP)-9. J Dermatol.

[8] Schiller PI, Itin PH (1996). Angiokeratomas: an update. Dermatology.

[9] Kim JH, Kim MR, Lee SH, Lee SE, Lee SH (2012). Dermoscopy: a useful tool for the diagnosis of angiokeratoma. Ann Dermatol.

[10] Jha AK, Sonthalia S, Jakhar D (2018). Dermoscopy of angiokeratoma. Indian Dermatol Online J.

[11] Bechara FG, Altmeyer P, Jansen T (2004). Unilateral angiokeratoma scroti: a rare manifestation of a vascular tumor. J Dermatol.

[12] Piqué-Duran E, Pérez-Cejudo JA, Cameselle-Martínez D, García-Vázquez O (2013). Unilateral angiokeratoma of Fordyce. Actas Dermosifiliogr.

[13] Pande SY, Kharkar D, Mahajan S (2004). Unilateral angiokeratoma of Fordyce. Indian J Dermatol Venereol Leprol.

[14] González-López MA, Consuegra G, Lacalle M, González-Vela MC (2017). Unilateral angiokeratoma of the scrotum (Fordyce's type) associated with a contralateral varicocele. Indian J Dermatol Venereol Leprol.

[15] Vinod A, Jacob P, Nair AT, Ranjith VB (2022). Unilateral angiokeratoma of Fordyce with unilateral PEAKER: a rare presentation of an uncommon disease. Cureus.

[16] Buljan M, Poduje S, Situm M, Bulat V, Bolanča Z, Tomas D (2010). Multiple angiokeratomas of the vulva: case report and literature review. Acta Dermatovenerol Croat.

[17] Doğan F, Bucak İH (2017). Rare giant angiokeratoma of the vulva: a case report. Balkan Med J.

[18] Samudrala S, Bhat MR (2018). Recurrent, nodular lesions over the vulva: a diagnostic challenge. Indian J Sex Transm Dis AIDS.

[19] Bjekić M, Marković M, Šipetić S (2012). Angiokeratoma of Fordyce in man and woman — case reports. Open Medicine.

[20] Leducq S, Giraudeau B, Tavernier E, Maruani A (2019). Topical use of mammalian target of rapamycin inhibitors in dermatology: a systematic review with meta-analysis. J Am Acad Dermatol.

